# A multicentre observational study of the prevalence, management, and outcomes of subsegmental pulmonary embolism

**DOI:** 10.1007/s11239-022-02714-5

**Published:** 2022-11-07

**Authors:** Michael N Armitage, Aishah Z Mughal, Christopher C Huntley, Daniel Lasserson, Michael Newnham

**Affiliations:** 1 University Hospitals Coventry and Warwickshire NHS Trust, Coventry, United Kingdom; 2 University of Birmingham, Birmingham, United Kingdom; 3 Occupational and Interstitial Lung Disease Services, Institute of Applied Health Research, University Hospitals Birmingham NHS Foundation Trust, University of Birmingham, Birmingham, United Kingdom; 4 University of Warwick, Coventry, United Kingdom; 5 Oxford University Hospitals NHS Foundation Trust, Oxford, United Kingdom; 6 Institute of Applied Health Research, University Hospitals Birmingham NHS Foundation Trust, University of Birmingham, Mindelsohn Way, B15 2GW Edgbaston, Birmingham, West Midlands United Kingdom

**Keywords:** Anticoagulants, Embolism, Prevalence, Pulmonary embolism, Venous thromboembolism

## Abstract

**Background:**

The incidence of subsegmental pulmonary embolism (SSPE) has increased with improvements in imaging technology. There is clinical equipoise for SSPE treatment, with conflicting evidence of improved mortality or reduced venous thromboembolism recurrence with anticoagulation. SSPE studies have significant heterogeneity and often lack adequately matched disease comparator groups.

**Objectives:**

To determine the prevalence, management, and outcomes of SSPE and compare them to patients with main, lobar, segmental, and no pulmonary embolism (PE).

**Patients/Methods:**

All adult patients undergoing CT pulmonary angiography (CTPA) between 2013 and 2019, at 3 UK hospitals were included in the study. CTPA reports were text mined for language relating to PE, and then further manually screened for the presence and anatomical location of PE. Patient groups were propensity matched by age, sex, and year of CTPA prior to analysis. 3-month outcomes of major bleeding, VTE recurrence, and death were recorded.

**Results:**

79 (3.8%) SSPEs were identified from 2,055 diagnoses of PE, and 14,300 CTPA reports. 44 (56%) of SSPEs were single artery emboli, 25 (32%) were multiple unilateral emboli, and 10 (13%) were multiple bilateral emboli. Mortality, VTE recurrence and major bleeding were similar at 3 months across all groups. 87.3% of SSPE imaging reports had an additional radiological diagnosis, with pleural effusion (30%), consolidation (19%), and cardiomegaly (19%) being the most common.

**Conclusion:**

The prevalence of SSPE was 3.8% of all PEs and there were a substantial number of additional radiological findings in the SSPE group that may have accounted for their symptoms.

**Supplementary Information:**

The online version contains supplementary material available at 10.1007/s11239-022-02714-5.

## Essentials

1. Equipoise remains regarding the management of subsegmental pulmonary embolism.
2. Observational study of three hospital sites within the United Kingdom.
3. Prevalence of SSPE in this population was 3.8% out of 2,055 PEs.
4. Additional radiological diagnoses were found in 87.3% of patients with SSPE.

## Introduction

Subsegmental pulmonary emboli (SSPE) affect the fourth division, or more distal pulmonary arterial branches. SSPE are difficult to identify on computed tomography (CT) scanning compared to more proximal PEs, with low interobserver agreement and a paucity of validated radiological criteria for their diagnosis [1–3].

The proportion of PEs diagnosed that are SSPE has increased with the use of advanced imaging techniques that assess the pulmonary vasculature. A 2010 systematic review and meta-analysis demonstrated a rise in the proportion of SSPE from 4.7% with single-detector CT to 9.4% using multi-detector CTs [4]. The proportion of SSPE varies from 4.5 to 11% of all PEs in retrospective observational studies that employ differing methodologies and diagnostic criteria [5–9]. A systematic review and meta-analysis of SSPE of 14 studies that included 15,563 diagnoses of PE, found 693 (4.6%) of these were SSPE (10). However, this analysis was limited by high study heterogeneity and a lack of control groups. In specific cohorts, the prevalence of SSPE has been estimated at 5.4% in post-operative orthopaedic patients, and 11% in older patients [10, 11]. A recent prospective study of symptomatic PE patients found an incidence of SSPE of 8.4% per year [12].

Despite the increased prevalence of SSPE there has not been a corresponding rise in mortality which may suggest overdiagnosis [13]. Patients with SSPE have a lower incidence of haemodynamic instability, right ventricular dysfunction, and simultaneous proximal deep vein thrombosis (DVT) [14, 15]. A Cochrane review concluded that there are currently no randomised controlled trial (RCT) reports on the effectiveness of anticoagulation in SSPE and consequently there is uncertainty about clinical management [16]. In practice, most physicians would offer anticoagulation to patients with SSPEs however, some studies have highlighted that a no-anticoagulation strategy would be considered particularly for single isolated SSPE (with no concurrent DVT) where the risk of VTE recurrence was low [17]. The decision to anticoagulate seems to be driven by the perceived risk of VTE recurrence, with physicians considering no-anticoagulation if the risk was less than 2% at 3 months [18]. In a recent study of patients with SSPE who did not have proximal DVT the incidence of VTE recurrence was 3.1%, however no fatalities were observed [19]. The treatment of SSPE in cancer is also contentious and whilst most physicians would choose to anticoagulate patients, there are differences in practice between specialities [20]. Retrospective studies of SSPE have also found variable management practices [5, 6, 12, 21–26].

Meta-analysis of outcomes demonstrated an 8.1% incidence of bleeding in SSPE patients treated with anticoagulation, with 3-month VTE recurrence rates of 5.3% in treated and 3.9% in untreated patients however, the wide and overlapping confidence intervals made it difficult to draw firm conclusions about group differences [27]. As VTE recurrence rates are similar for treated and untreated patients, the decision to use anticoagulation to prevent VTE recurrence should be balanced against bleeding risks. Major bleeding and clinically relevant non-major bleeding (CRNMB) are the standardised definitions applied, but they are not consistently utilised across SSPE studies, which makes it challenging to compare bleeding rates in SSPE with other PE distributions. In the same meta-analysis, the 3-month mortality rate was 2.1% in anticoagulation treated SSPE patients compared to 3.0% in no-anticoagulation patients [27].

In the current study, we investigate the prevalence, risk factors, additional radiological findings and 3-month outcomes (VTE recurrence, bleeding, mortality) in patients with SSPE using standardised definitions and compare them to propensity matched groups with no PE and more proximal PEs.

## Methods

### Patient selection & study design

The study was approved by the NHS Health Research Authority (HRA) (REC reference 21/PR/1443). All consecutive CT pulmonary angiogram (CTPA) reports from scans performed between 01/01/2013 and 01/01/2019 at three United Kingdom (UK) hospitals were screened for inclusion in the study. Reports from patients under 18 years of age or diagnosed with chronic thromboembolic disease were excluded from the analysis. CTPA reports were text mined (R statistical software (v3.5.1)) for wording related to pulmonary embolism [28]. All CTPA reports identified with a PE present, were manually screened for confirmation of the diagnosis and anatomical location of the PE (main/central, lobar, segmental, subsegmental) was recorded. PE groups were determined by the most proximally reported embolic material within the pulmonary arteries (PA). Patients with SSPE were then propensity matched (nearest neighbour method) by age, sex, and year of CTPA to patients without PE, and to patients with PE in other anatomical PA distributions (main/central, lobar, or segmental PE) for inclusion in the final analysis.

After study groups were defined, patient electronic health records were reviewed for data on demographics, comorbidities, admission details, VTE risk factors, blood markers (d-dimer, troponin, haemoglobin), lower limb compression and doppler ultrasound, chosen anticoagulation treatment regime, and clinical outcomes. Pre-existing anticoagulation refers to the regular use of any form of anticoagulation at time of PE diagnosis. Data was anonymised and processed prior to statistical analysis.

### Clinical outcomes

Clinical outcomes for significant bleeding events, VTE recurrence, and mortality were assessed within 3 months following the date of diagnosis of pulmonary emboli. Bleeding events were categorised using criteria for non-surgical patients established by the International Society on Thrombosis and Haemostasis (ISTH). Major bleeding was defined as patients with symptomatic presentation alongside fatal bleeding, bleeding from an organ or critical anatomical area, and/or bleeding resulting in a decrease in serum haemoglobin levels by at least 20gL^− 1^ or requiring a transfusion of ≥ 2 units of whole blood or red cells [29].

VTE recurrence was defined as all episodes of acute pulmonary thromboembolism or DVT within 3 months of the date of diagnosis of pulmonary emboli. All cases were radiologically confirmed using either CTPA, ventilation-perfusion (VQ) scan, or ultrasound of the lower limbs. Survival status was determined as patient death within 3 months following the date of diagnosis of pulmonary emboli. An NHS summary care record tracking system was also reviewed to assess patient survival status at time of follow-up.

### Additional radiological diagnoses

Alongside reviewing CTPA reports to confirm the diagnosis of pulmonary emboli, all CTPA reports were assessed for additional radiological diagnoses and technical adequacy of CTPA images. Relevant recorded diagnoses included the presence of pleural effusion, consolidation, emphysema, lung cancer, pulmonary fibrosis, ground glass opacity, bronchiectasis, cardiomegaly, and other radiological diagnoses. Other radiological diagnoses included mediastinal/hilar lymphadenopathy, lung collapse, pulmonary oedema, and acute rib fractures.

### Statistical analysis

Statistical analysis was performed using the statistical software R (v3.5.1) [28]. Baseline characteristics and clinical outcomes were presented as frequency and percentages for discrete variables and median ± interquartile range (IQR) for continuous data which was non-normally distributed. Differences in clinical outcomes between comparator groups were evaluated using Fisher’s exact test and Mann-Whitney U test for discrete and continuous data, respectively, to calculate *p*-values. A *p*-value of < 0.05 was deemed statistically significant.

## Results

### Cohort characteristics

**Fig. 1:**
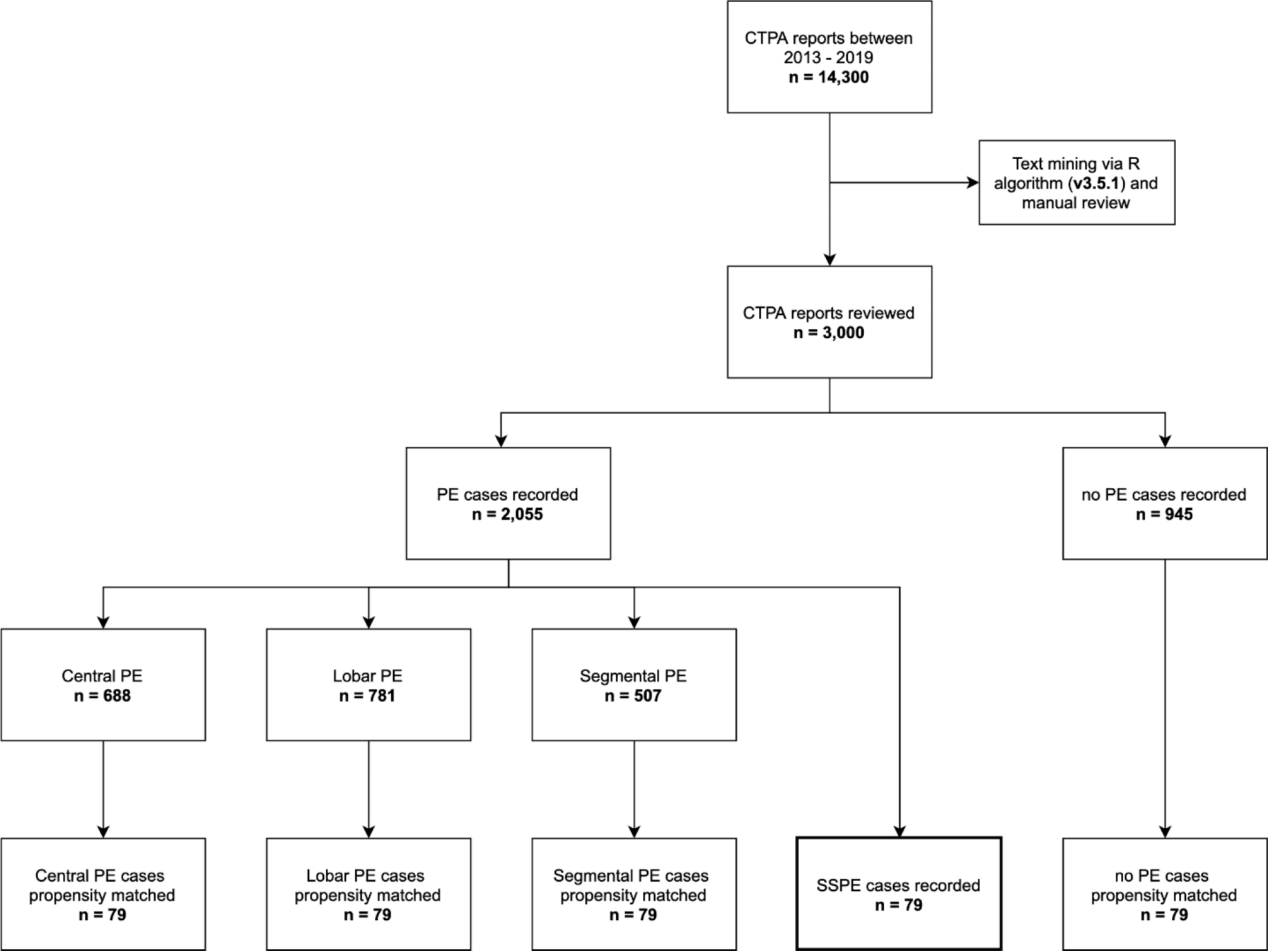
Flowchart of the data collection process. Propensity matching was performed according to age, sex, and year of CTPA. CTPA, computed tomographic pulmonary angiogram; SSPE, subsegmental pulmonary emboli; PE, pulmonary emboli

In total, 2,055 (14.4%) diagnoses of PE were made from 14,300 CTPAs. Of those patients with PE, 79 (3.8%) were SSPE, 507 (24.7%) were segmental, 781 (38.0%) were lobar, and 688 (33.5%) were main/central PE (Fig. 1).

Baseline characteristics of subsegmental, segmental, lobar, main, and no PE groups are summarised in Table 1. Age, sex, and year of CTPA were not significantly different across groups due to propensity matching. There were also no significant differences in admission duration or time to CTPA between groups. There was a trend towards patients having more active cancer in the SSPE (26.6%), segmental PE (33.3%), and lobar PE groups (30.4%), compared with 19% in the no PE group, but the difference was not seen in the main group (16.5%). The main PE group had the highest levels of serum troponin (median 100 ng/L [IQR 21–366]) and D-dimer (median 2590 ng/mL [IQR 1525–4672]) compared with the other groups. Missingness of data has been summarised in Table S1 in the supplementary materials.

**Table 1:** Baseline characteristics of SSPE, segmental, lobar, main, and no PE groups. CTPA, computed tomography pulmonary angiogram; USS, ultrasound; DOAC, direct oral anticoagulant; LMWH, low molecular weight heparin. Data are presented as n (%) unless stated otherwise. See Supplementary Table 1 for individual data N values. *The other anticoagulation duration category included durations less than 3 months

	SSPE (n = 79)	Segmental PE (n = 79)	Lobar PE (n = 79)	Main PE (n = 79)	No PE (n = 79)
**Sex (male)**	42 (53.2)	46 (58.2)	43 (54.5)	43 (54.5)	41 (51.9)
**Age (years), median [IQR]**	71 [22]	71 [21]	72 [24]	73 [19]	67 [23]
**Active cancer**	21 (26.6)	26 (33.3)	24 (30.4)	13 (16.5)	15 (19.0)
**Pregnant**	1 (1.3)	0 (0.0)	1 (1.3)	0 (0.0)	0 (0.0)
**Comorbidities**					
**–Hypertension**	27 (34.2)	17 (21.5)	22 (27.8)	31 (39.2)	33 (41.8)
** Diabetes mellitus**	11 (13.9)	10 (12.7)	7 (8.9)	15 (19.0)	18 (22.8)
** Asthma/COPD**	19 (24.1)	20 (25.3)	13 (16.5)	16 (20.3)	19 (24.1)
** Atrial fibrillation**	8 (10.1)	4 (5.1)	7 (8.9)	2 (2.5)	5 (6.3)
** Ischaemic heart disease**	11 (13.9)	7 (8.9)	11 (13.9)	10 (12.7)	9 (11.4)
**Year of CTPA, median [IQR]**	2016 [3]	2016 [2]	2017 [2]	2016 [2]	2016 [3]
**Time to CTPA (days), median [IQR]**	1 [4]	1 [2]	1 [3]	1 [2]	1 [3]
**Admission duration (days), median [IQR]**	4 [12]	4 [27]	4 [9]	6 [6]	4 [13]
**D-dimer (ng/mL), median [IQR]**	644 [744]	1025 [1687]	1006 [2458]	2590 [3147]	390 [1068]
**Troponin (ng/L), median [IQR]**	10 [29]	12 [30]	10 [10]	100 [345]	10 [56]
**Ultrasound of lower limb**	2 (2.6)	3 (3.9)	10 (12.7)	9 (1.4)	11 (13.9)
**Previous anticoagulation**	15 (19.0)	10 (12.7)	5 (6.3)	1 (1.3)	7 (8.9)
**Anticoagulation prescribed**	75 (96.2)	73 (98.6)	77 (97.5)	78 (98.7)	3 (3.8)
**Anticoagulation type**					
** None**	3 (3.8)	1 (1.4)	2 (2.5)	1 (1.3)	76 (96.2)
** LMWH**	18 (24.0)	17 (23.0)	19 (24.1)	10 (12.7)	0 (0)
** Warfarin**	13 (17.3)	13 (17.6)	17 (21.5)	20 (25.3)	0 (0)
** DOAC**	43 (57.3)	43 (58.1)	41 (51.9)	48 (60.8)	3 (3.8)
**Anticoagulation duration**					
** Admission**	3 (4.3)	1 (1.4)	0 (0.0)	1 (1.3)	0 (0)
** 3 months**	8 (11.4)	7 (10.1)	2 (2.6)	5 (6.4)	0 (0)
** 6 months**	13 (18.6)	14 (20.3)	18 (23.4)	27 (34.6)	0 (0)
** Long-term**	36 (51.4)	39 (56.5)	35 (45.5)	30 (38.5)	3 (100)
Other*	10 (14.3)	8 (11.6)	22 (28.6)	15 (19.2)	0 (0)

The median age of patients with SSPE was 71 [IQR 59–82] years and 53% were male. 21 SSPE patients (26.6%) had active cancer and 1 patient was pregnant (1.3%) at the time of diagnosis. Only 2 patients underwent ultrasound assessment of the lower limbs for DVT. Following SSPE diagnosis, 96.2% of patients were treated with anticoagulation therapy that was predominately direct oral anticoagulants (DOACs) (57.3%) for 3–6 months (30%) or long-term (51.4%). The no PE patients were an appropriate control group but did not constitute a healthy cohort with 19% having active cancer and 32.9% previously treated with anticoagulation therapy prior to admission.

### Clinical outcomes

There were no differences in the 3-month outcomes of VTE recurrence, major bleeding, or mortality across all groups (Table 2). VTE recurrence was less than 3% across all comparator groups, and zero in the lobar, SSPE, and no PE groups. All recorded outcomes were uniformly low across all groups.

**Table 2:** 3-Month outcomes in SSPE, segmental, lobar, main, and no PE groups. Data are presented as n (%). VTE, venous thromboembolism

	SSPE	Segmental PE	Lobar PE	Main PE	No PE
**VTE recurrence**	0 (0.0)	2 (2.6)	0 (0.0)	1 (1.3)	0 (0.0)
**Major bleeding**	3 (3.8)	3 (3.8)	3 (3.8)	2 (2.5)	3 (3.8)
**Mortality**	1 (1.3)	0 (0.0)	0 (0.0)	2 (2.5)	2 (2.5)

### Radiological findings

Of the 79 SSPEs diagnosed, 44 (56%) were a single embolus, 10 (13%) were multiple bilateral emboli, 19 (24%) were multiple unilateral emboli (single lobe), 5 (6%) were multiple unilateral emboli (two lobes), and 1 (1%) was multiple unilateral emboli (three lobes). Additional radiological findings extracted from CTPA reports are summarised in Table 3. CTPA scans were deemed technically adequate by the reporting radiologist in > 95% of cases across all groups. Patients diagnosed with SSPE had the highest overall proportion of additional radiological diagnoses (87.3%) whilst those with main PE had the lowest proportion of additional radiological findings (60.8%). The three most identified additional radiological diagnoses were pleural effusion, consolidation, and emphysema.

**Table 3:** Additional CTPA radiological findings in SSPE, segmental, lobar, main, and no PE groups. Data is presented as n (%)

		SSPE (n = 79)	Segmental PE (n = 79)	Lobar PE (n = 79)	Main PE (n = 79)	No PE (n = 79)
**Technically adequate**		77 (97.5)	79 (100.0)	77 (97.5)	79 (100.0)	76 (96.2)
**Additional radiological findings**		69 (87.3)	58 (73.4)	58 (73.4)	48 (60.8)	67 (84.8)
** Pleural effusion**		24 (30.4)	19 (24.1)	32 (40.5)	11 (13.9)	20 (25.3)
** Consolidation**	15 (19.0)	21 (26.6)	26 (32.9)	16 (20.3)	22 (27.8)	
** Emphysema**	13 (16.5)	21 (26.6)	12 (15.2)	17 (21.5)	17 (21.5	
** Lung cancer**	8 (10.1)	5 (6.3)	7 (8.9)	3 (3.8)	6 (7.6)	
** Pulmonary fibrosis**	7 (8.9)	2 (2.5)	0 (0.0)	6 (7.6)	9 (11.4)	
** Ground glass opacity**	4 (5.1)	8 (10.1)	13 (16.5)	14 (17.7)	14 (17.7)	
** Bronchiectasis**	5 (6.3)	6 (7.6)	4 (5.1)	5 (6.3)	14 (17.7)	
** Cardiomegaly**	15 (19.0)	4 (5.1)	6 (7.6)	5 (6.3)	9 (11.4)	
**Other radiological diagnoses** ^**¶**^		18 (22.8)	15 (19.0)	24 (30.4)	13 (16.5)	22 (27.8)

^¶^Other radiological diagnoses include lymphadenopathy, collapse, pulmonary oedema and rib fractures. Patients may have more than one radiological diagnosis recorded

## Discussion

This multicentre observational study investigated the prevalence, risk factors, additional radiological findings, and outcomes of patients with SSPE over six years across three UK hospitals. Of 14,300 CTPA reports screened, 14.4% had a diagnosis of PE and of these 3.8% had a diagnosis of SSPE. There was no significant difference found across all groups in mortality, VTE recurrence or major bleeding at 3 months. This is the first SSPE study to include propensity-matched patients with more proximal or no PE diagnoses. This is important for comparing outcomes across PE groups that can vary by other factors (i.e., age, sex).

The prevalence of SSPE in observational studies has been reported to be as high as 13.5%, whereas our current study prevalence of 3.8% is similar to the pooled estimate of 4.6% (95% confidence interval 1.8-8.5%) in a meta-analysis [9, 27]. The 3-month SSPE outcomes in the current study were 0% for VTE recurrence, 3.8% for major bleeding and 1% for mortality. The uniformly low outcome frequency across all groups limited comparisons between SSPE and more proximal or no PE patients. A recent study by Ceccato et al., also reported low 3-month outcomes with no cases of VTE recurrence in either anticoagulant treated or untreated SSPE patients [12]. This compares with 5.3% VTE recurrence, 8.1% bleeding, and 2.1% mortality for the anticoagulation treated SSPE patients in the Bariteau et al., meta-analysis [10]. However, the studies included in this meta-analysis were heterogenous with many lacking standardised bleeding definitions and utilised a combination of major bleeding and CRNMB that may explain the discordance in bleeding rates. Our study primarily used secondary care hospital records and therefore we were unable to report on the CRNMB episodes.

As the majority (96.2%) of SSPE patients were treated with anticoagulation there were insufficient untreated patients to make comparisons between the two groups. The American College of Chest Physicians (ACCP) guidelines on antithrombotic therapy for VTE disease recommend that SSPE can be treated with anticoagulation or no-anticoagulation (surveillance) depending on the patient’s VTE risk profile [31]. If a no-anticoagulation strategy is considered, then bilateral lower limb ultrasound (or alternative imaging) should be performed, as the presence of proximal DVT would necessitate anticoagulation. Previous studies have either not reported the number of patients investigated for DVT [32], or reported incomplete rates of investigation [7]. Fernández-Capitán et al. report that 55% of patients with SSPE did not have a concurrent DVT, however not all patients in this study were investigated for DVT [7]. Interestingly, they also found that the VTE-recurrence rates were similar in SSPE patients with and without DVT.

International surveys of thrombosis experts have highlighted the clinical equipoise in SSPE management and to date no RCTs have compared anticoagulation to surveillance [17, 18]. The use of anticoagulation in VTE is not without risk and treatment with DOACs is associated with major bleeding in 1.1% and CRNMB in 6.6% of patients during pooled follow-up of 3–6 months in a meta-analysis [33]. The risk of bleeding in anticoagulant treated SSPE has been reported as 8.1%, and in other instances of untreated VTE it is 0.6% per patient year [27, 30]. In a recent large prospective cohort study of patients with untreated SSPE by Le Gal et al., the 90 day risk of major bleeding was found to be 0.7%, and minor bleeding 1.4% [19]. Unsurprisingly, the risk of major bleeding is higher in our study (3.8%) where most patients with SSPE were treated with anticoagulation. The reported rate of VTE recurrence was higher in the Le Gal study compared to the present study, with 3.1% recurrence within 90 days compared to 0% respectively. This may reflect more standardised monitoring and recording of VTE events during the follow-up period compared to a retrospective cohort. Despite the difference in anticoagulation treatment the mortality rates for both studies were similar, at 1.4% in the Le Gal study and 1.3% in the current study. It remains unclear if anticoagulation in SSPE results in a net-benefit of improved VTE recurrence compared with the bleeding risks. There are ongoing multicentre SSPE RCTs in the UK (STOP-APE; stopping anticoagulation for isolated SSPE [NCT04727437]), and Canada / Switzerland and the Netherlands (NCT04263038; Surveillance vs. Anticoagulation For low-risk patiEnts with isolated SubSegmental Pulmonary Embolism) [34, 35] to address this important question.

The additional radiological findings were highest (87.3%) in the SSPE group, with the most common diagnoses being pleural effusion, consolidation, and cardiomegaly. Whilst consolidation and cardiomegaly could be a consequence of lung infarction and right heart strain associated with PE, this is less likely with smaller embolic loads. Therefore, the symptoms that precipitated investigation for PE may have been driven by alternative pathology and the SSPEs may have been contributory or incidental findings. The majority of CTPAs in the SSPE group were considered technically adequate by the reporting radiologists, which is important as SSPE can be challenging to diagnose with greater false positives than more proximal PEs [36]. This could result in overtreatment of non-existent SSPEs and the potential harm from anticoagulant therapy. The VTE guidelines from the ACCP proposed clinical and diagnostic criteria that may make a diagnosis of SSPE more likely however, this was based on expert consensus and is yet to be validated in clinical trials [31]. One of these criteria is the presence of PE related symptoms, however in our current study there is a high incidence of additional radiological findings in the SSPE, and the no PE groups, suggesting some symptoms may be driven by alternative diagnoses. Furthermore, the European Society of Cardiology guidelines on acute pulmonary embolism (2019) advocate discussing single SSPEs with a radiologist and if necessary, seeking a second opinion to confirm the diagnosis [37].

The strengths of the current study are that it (i) included patients across several hospitals over a six-year period, (ii) utilised propensity matching between SSPE and main, lobar, segmental, and no PE groups, (iii) identified cases from CTPA reports which is more robust than clinical coding and, (iv) included standardised definitions of 3-month outcomes from VTE recurrent and major bleeding. There are several limitations to the current study. Firstly, PE and SSPE cases were identified from CTPA reports but were not all reviewed by a thoracic radiologist. There is significant inter-observer variation in the diagnosis of SSPE between general and thoracic radiologists and the number of false positives is reduced by increasing specialisation [38]. However, given the ubiquity and frequency of CTPAs, thoracic radiology review of all scans is often not performed at all institutions and therefore the current study represents the current real-world practice. Secondly, only CTPA reports were utilised for case finding, which did not capture PEs identified by other modalities including ventilation/perfusion (VQ) scans. This is likely to have had a negligible effect on prevalence estimates as CTPA was the first line investigation for PE at the study sites and VQ was not widely performed. However, it is possible that a number of PEs were identified on other CTs such as staging CT scans and these have not been included in this study. Thirdly, propensity matching for other factors that may have influenced PE outcomes including active cancer was not possible as this information was only available following case note reviews. Fourthly, there may have been further factors that influenced PE outcomes in addition to embolus anatomical location including the embolic load, degree of right heart strain, presence of concurrent acute illness, and level of pre-existing physiological reserve. Finally, the use of electronic patient records alone for follow-up data may have underestimated some outcomes that are not immediately recorded in hospital records. One instance of this may be reflected in mortality which is lower than expected for more central PEs. Alternatively, this may reflect lower mortality for patients with PE in more modern cohorts.

In conclusion, this multicentre observational study screened 14,300 CTPA reports to identify 2,055 (15%) PEs, of which 79 (3.8%) were SSPEs. There were a substantial number of additional radiological findings in the SSPE group that may have accounted for their symptoms and contributes to the challenge of balancing the risks and benefits of anticoagulation. There remains clinical equipoise in the management of SSPE that will be addressed by ongoing RCTs.

## Electronic supplementary material

Below is the link to the electronic supplementary material.

Supplementary Material 1
