# Correction to: A multicentre observational study of the prevalence, management, and outcomes of subsegmental pulmonary embolism

**DOI:** 10.1007/s11239-023-02771-4

**Published:** 2023-01-20

**Authors:** Michael N. Armitage, Aishah Z. Mughal, Christopher C. Huntley, Daniel Lasserson, Michael Newnham

**Affiliations:** 1 University Hospitals Coventry and Warwickshire NHS Trust, Coventry, UK; 2 University of Birmingham, Birmingham, UK; 3 Occupational and Interstitial Lung Disease Services, Institute of Applied Health Research, University Hospitals Birmingham NHS Foundation Trust, University of Birmingham, Birmingham, UK; 4 University of Warwick, Coventry, UK; 5 Oxford University Hospitals NHS Foundation Trust, Oxford, UK; 6 Institute of Applied Health Research, University Hospitals Birmingham NHS Foundation Trust, University of Birmingham, Mindelsohn Way, B15 2GW Edgbaston, Birmingham, West Midlands UK

**Correction to: Journal of Thrombosis and Thrombolysis (2022)** https://doi.org/10.1007/s11239-022-02714-5

In the original version of the article:

1. The name of Aishah Z Aishah (2) has been duplicated for both name and surname; the correct author name is “Aishah Z Mughal”

These have been corrected with this erratum.

