# Supplementary material for: A multicentre observational study of the prevalence, management, and outcomes of subsegmental pulmonary embolism
Source: J Thromb Thrombolysis. 2022 Nov 7;55(1):126–33. doi: 10.1007/s11239-022-02714-5 (PMC9925472; doi:10.1007/s11239-022-02714-5)
Supplement: Supplementary file 1 — Supplementary Material 1 [file 11239_2022_2714_MOESM1_ESM.docx]

**Supplementary Materials**

| **Supplementary Table 1.** Table of available data by variable. | | | | | |
| --- | --- | --- | --- | --- | --- |
|  | **SSPE** | **Segmental** | **Lobar** | **Main** | **No PE** |
| **Date of birth** | 78 | 79 | 79 | 79 | 79 |
| **Year of CTPA** | 79 | 79 | 79 | 79 | 79 |
| **Age** | 78 | 79 | 79 | 79 | 79 |
| **Sex** | 79 | 79 | 79 | 79 | 79 |
| **Cancer diagnosis** | 79 | 78 | 79 | 79 | 79 |
| **Pregnancy** | 77 | 78 | 79 | 79 | 79 |
| **Time to discharge** | 79 | 74 | 78 | 77 | 79 |
| **Time to CT** | 79 | 76 | 79 | 79 | 79 |
| **D-dimer** | 37 | 28 | 45 | 45 | 29 |
| **Troponin** | 27 | 22 | 29 | 47 | 23 |
| **Ultrasound of lower limb** | 78 | 77 | 79 | 79 | 79 |
| **Previous anticoagulation** | 79 | 79 | 79 | 79 | 79 |
| **Anticoagulation started** | 78 | 74 | 79 | 79 | 78 |
| **Anticoagulation type** | 75 | 74 | 79 | 79 | 66 |
| **Anticoagulation duration** | 71 | 71 | 79 | 79 | 67 |
| **VTE recurrence** | 79 | 78 | 79 | 79 | 79 |
| **Major bleeding** | 79 | 78 | 79 | 79 | 79 |
| **Death** | 79 | 79 | 79 | 79 | 79 |
